# Cooperative polariton dynamics in feedback-coupled cavities

**DOI:** 10.1038/s41467-017-01796-7

**Published:** 2017-11-10

**Authors:** Bimu Yao, Y. S. Gui, J. W. Rao, S. Kaur, X. S. Chen, W. Lu, Y. Xiao, H. Guo, K. -P. Marzlin, C. -M. Hu

**Affiliations:** 1 Department of Physics and Astronomy, University of Manitoba, Winnipeg, Manitoba Canada R3T 2N2; 2 State Key Laboratory of Infrared Physics, Chinese Academy of Sciences, Shanghai, 200083 China; 3 College of Science, Nanjing University of Aeronautics and Astronautics, Nanjing, 210016 China; 4 Department of Physics, McGill University, Montréal, Québec Canada H3A 2T8; 5 Department of Physics, St. Francis Xavier University, Antigonish, Nova Scotia Canada B2G 2W5; 6 Department of Physics and Atmospheric Science, Dalhousie University, Halifax, Nova Scotia Canada B3H 4R2

## Abstract

The emerging field of cavity spintronics utilizes the cavity magnon polariton (CMP) induced by magnon Rabi oscillations. In contrast to a single-spin quantum system, such a cooperative spin dynamics in the linear regime is governed by the classical physics of harmonic oscillators. It makes the magnon Rabi frequency independent of the photon Fock state occupation, and thereby restricts the quantum application of CMP. Here we show that a feedback cavity architecture breaks the harmonic-oscillator restriction. By increasing the feedback photon number, we observe an increase in the Rabi frequency, accompanied with the evolution of CMP to a cavity magnon triplet and a cavity magnon quintuplet. We present a theory that explains these features. Our results reveal the physics of cooperative polariton dynamics in feedback-coupled cavities, and open up new avenues for exploiting the light–matter interactions.

## Abstract (web summary)

Rabi oscillations of magnons typically do not have few photon control of single spin quantum systems. Here, the authors use a feedback cavity architecture to increase magnon-photon cooperativity, enabling increased control of light-matter interactions in magnonic systems via cooperative polariton dynamics.

## Introduction

Controlling cooperativity is essential for utilizing the light–matter interactions^1^. For light coupled with quantum systems, cooperativity measures the ratio of Rabi frequency to decay rates and can be controlled by changing the photon Fock state occupation^2^. Recently, high cooperativity was demonstrated in hybridizing magnons and cavity microwave photons^3–10^. In contrast to a single spin system^2^, the harmonic oscillator nature of the ensemble of spins in the linear dynamic regime makes the magnon Rabi frequency independent of the photon number^11–14^. Breaking the harmonic limit has been a pursuit of continuing interest in the study of collective dynamics, as, for example, in the case of the Kohn theorem^15–17^. The same quest is developing in the new field of cavity spintronics^18^, where it is required for quantum control of collective spin dynamics^19, 20^.

Here we show that a feedback cavity architecture breaks the harmonic limit for magnon–photon coupling. By increasing the voltage-controlled feedback photon number, we observe an increase of the Rabi frequency, accompanied with the evolution of the cavity magnon polariton^7, 11, 12^ to a cavity magnon triplet and a cavity magnon quintuplet. The unprecedented capability of on-chip control of magnon-photon cooperativity, which our theory links to the physics of cooperative polariton dynamics, may open up exciting opportunities for exploiting the light–matter interactions.

## Results

### Device architecture

**Fig. 1:**
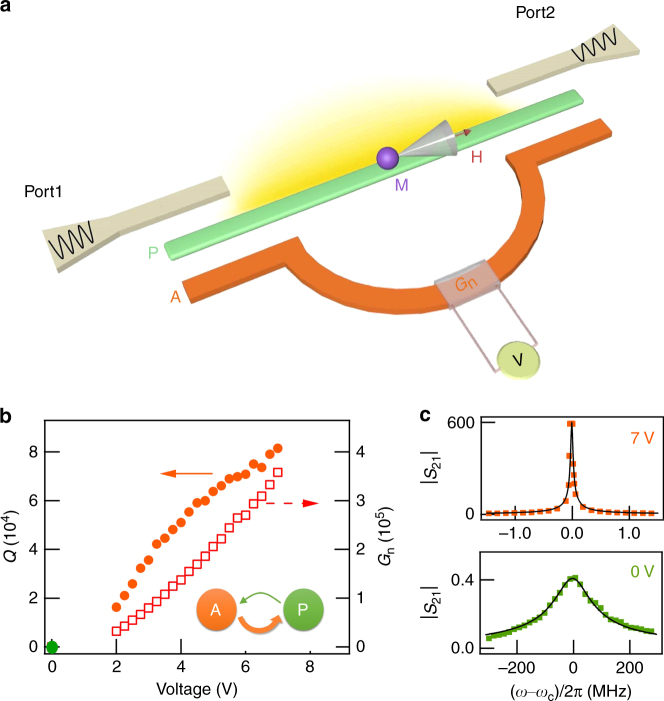
Device architecture. **a** The A-P-M device consists of a passive cavity (P), an active cavity **A** with a voltage tuneable gain (*G*_*n*_), and an YIG sphere with magnons (M). **b** The *Q*-factor and *G*_*n*_ of the A-P cavity circuit are tunable up to 81,500 and 360,000, respectively, by changing *V*. **c**, |*S*_21_| spectra of the cavity mode measured at *V* = 0 and 7 V, together with a fitted theoretical curve

Our experimental setup is schematically shown in Fig. 1a. We use a planar passive cavity (P) with a mode frequency *ω*_*c*_ to couple photons with the magnons (M) in a ferromagnetic insulator yttrium iron garnet (YIG). The YIG sphere is placed on top of the cavity at an adjustable distance *d*. An external magnetic field **H** is applied parallel to the cavity to tune the magnon frequency *ω*_*r*_. When *ω*_*r*_ approaches *ω*_*c*_, the magnon–photon coupling^21^ produces the cavity magnon polariton (CMP)^7^ in the P–M device, which is detected by using a two-port vector network analyzer (VNA) that measures the transmission coefficient *S*_21_(*ω*) of the P-cavity. The key innovation of our experiment, in comparison with the recent pioneering studies of magnon–photon coupling^3–10^, is the design and implementation of an active cavity (A), which contains a microwave amplifier with voltage (V)-controlled gain (*G*_*n*_). The two cavities (A and P) are carefully designed to have the same mode frequency. They are weakly coupled and phase matched to each other (see Supplementary Notes 1–3 for details). Such a feedback-coupled cavity architecture is crucial as it offers more capabilities than the versatile double beam technique^22^: Essentially, the A-cavity acts as a feedback loop to compensate the microwave loss of the P-cavity, and produces a gain up to 360,000; the cavity quality factor is tunable up to 81,500, about three orders of magnitude higher than conventional planar cavities. Furthermore, feedback photons couple with the CMP, and thus coherently enhance the strong coupling in the P-M device. Altogether, these capabilities reveal a hidden cooperative effect of polariton dynamics, which has escaped studies ever since 1954, when Dicke elucidated the cooperative molecular dynamics^23^ that led to the development of super-radiant devices^24, 25^.

A dozen A-P-M devices with different geometries were designed and showed consistent results, with a variety of new effects involving the light–matter interactions being revealed. In this paper the emphasis is on data rendered from a typical device, which focuses on the fundamental issue of the breaking of the harmonic protection of magnon-photon cooperativity.

The magnon mode we study has Gilbert damping parameter *α* = 2.0 × 10^−4^, and the dispersion follows the Kittel formula $\omega _r = \gamma \mu _0\left( {\left| H \right| + H_A} \right)$. Here, *γ* = 173 GHz T^−1^ is the electron gyromagnetic ratio, *μ*_0_ *H*_*A*_ = 8.56 mT is the effective field^3–10^, and *μ*_0_ is the permeability of vacuum. We study the A-P-M devices by using three methods to systematically control the CMP: (i) tuning *d* to control the P-M coupling strength, (ii) tuning *V* to control the feedback photon number, and (iii) tuning *H* to change the frequency detuning $\Delta \equiv \omega _r - \omega _c$.

### Distance *d* dependent Rabi frequency

**Fig. 2:**
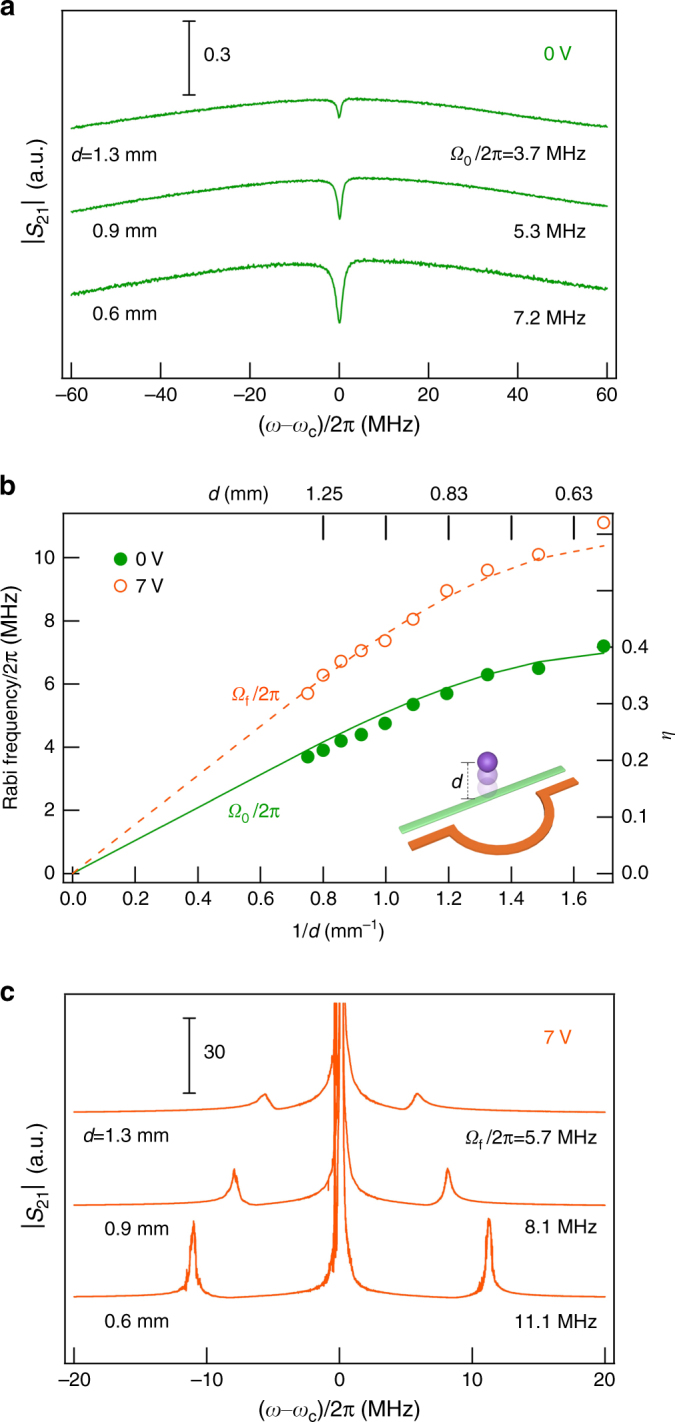
Distance dependent Rabi frequency. **a** |*S*_21_| spectra measured at Δ = 0 and *V* = 0 V, for *d* = 1.3, 0.9, and 0.6 mm. The corresponding Rabi frequencies *Ω*_0_ are determined by line shape fitting. **b** Rabi frequency as a function of *d*. The solid (open) circles represent data measured at *V* = 0 V (7 V). The solid curve represents *Ω*_0_/2*π* calculated from Eq. (1) by using η determined from a Maxwell’s equations solver, where the dashed curve is calculated with *Ω*_*f*_ = 1.5*Ω*_0_. **c** |*S*_21_| spectra measured at Δ = 0 and *V* = 7 V. The Rabi frequencies *Ω*_*f*_ are determined from the mode splitting

We begin our study with the feedback switched off (*V* = 0) and with an intra-cavity photon number of about 10^6^. Figure 2a shows the |*S*_21_| spectra measured at zero detuning (Δ = 0). Within the broad cavity mode of *Q* = 25, a dip appears at *ω*_*c*_/2*π* = 3.160 GHz. This is the characteristic spectral response of CMP in the regime of magnetically induced transparency^5^ defined by $1/2 Q  >\Omega_0 / \omega_c  >\alpha$. The Rabi frequency *Ω*_0_ depends on *d* since the rf field of the P-cavity increases with decreasing *d*. By fitting the |*S*_21_| spectra using the CMP theory^5, 11, 12^, we deduce *Ω*_0_ and plot the *Ω*_0_−*d* dependence in Fig. 2b (the solid circles).

The expected value of *Ω*_0_ is given by^3–5, 12, 21^

1 $$\Omega _0 = g_0\sqrt N ,$$

where $g_0 = \eta \gamma \sqrt {5\mu _0\hbar \omega _c/4V_c}$ is the vacuum Rabi frequency of a single spin, *N* = 1.1 × 10^19^ is the total number of spins in the YIG sphere, and *V*_*c*_ is the cavity mode volume. $\eta = h\left( d \right)/h\left( 0 \right)$ describes *d*-dependent spatial overlap between the rf field *h* and the magnon mode in YIG^5^, which we calculate by using a Maxwell’s equations solver. With *V*_*c*_ = 7.98 × 10^−5^ m^3^ as the only fitting parameter, the calculated Rabi frequency is plotted in Fig. 2b (solid curve), and agrees well with the measured result.

Eq. (1) shows that *Ω*_0_ has an $\sqrt N$-fold enhancement from *g*_0_. It is this cooperative spin dynamic effect^14, 21, 23, 26^ that makes cavity magnon systems highly interesting^3–10^. However, such a collective dynamics has a drawback: a single quantum two-level system can be saturated by one photon excitation, so that adding photons enhances the Rabi frequency^2^, but the magnon-photon coupling exhibits classical behavior^7^ of coupled harmonic oscillators^11, 12^. In the linear dynamic regime where the number of CMP $m \ll N$, the spin ensemble is far from being saturated, so that adding photons may increase *m* but does not change *Ω*_0_. Hence, in contrast to quantum systems^2^, the magnon-photon cooperativity, $C \equiv 2(\Omega _0/\omega _c)^2Q/\alpha$, has been found to be independent of photon number^7^. Breaking such a harmonic limit without interfering with the complex nonlinear magnetization dynamics^21, 27, 28^ seems impossible. But, as we reveal below, a distinct cooperative polariton dynamics may be used in the linear dynamic regime ($m \ll N$), which enables tuning of the magnon Rabi frequency by the changing of the feedback photon numbers.

### Feedback dependent Rabi frequency

Figure 2c shows the |*S*_21_| spectra still measured at Δ = 0, but with the amplifier switched on at *V* = 7 V. Feedback gain of *G*_*n*_ = 3.6 × 10^5^ with a feedback photon number of *n* = 1 × 10^13^ is created. The cavity mode frequency shifts slightly to *ω*_*c*_/2*π* = 3.177 GHz, while the *Q*-factor is significantly increased to 81,500. Noticeably, the A-P-M device enters the strong coupling regime^3–10^, where $\Omega_f / \omega_c  >\alpha  >1/2 Q$. Here, *Ω*_*f*_ is the Rabi frequency enhanced by the feedback photons, and is deduced from the two side peaks at *ω*_*c*_±*Ω*_*f*_. As shown in Fig. 2b, both *Ω*_*f*_ and *Ω*_0_ increase with decreasing *d*, but the ratio of *Ω*_*f*_/*Ω*_0_ = 1.5 stays constant. Another remarkable feature is that a sharp resonance appears in the spectral center at *ω*_*c*_, indicating that the twin CMP evolve to a cavity magnon triplet. It resembles the Mollow triplet^29^, a canonical signature of the light–matter interaction observed in single quantum systems^22^.

**Fig. 3:**
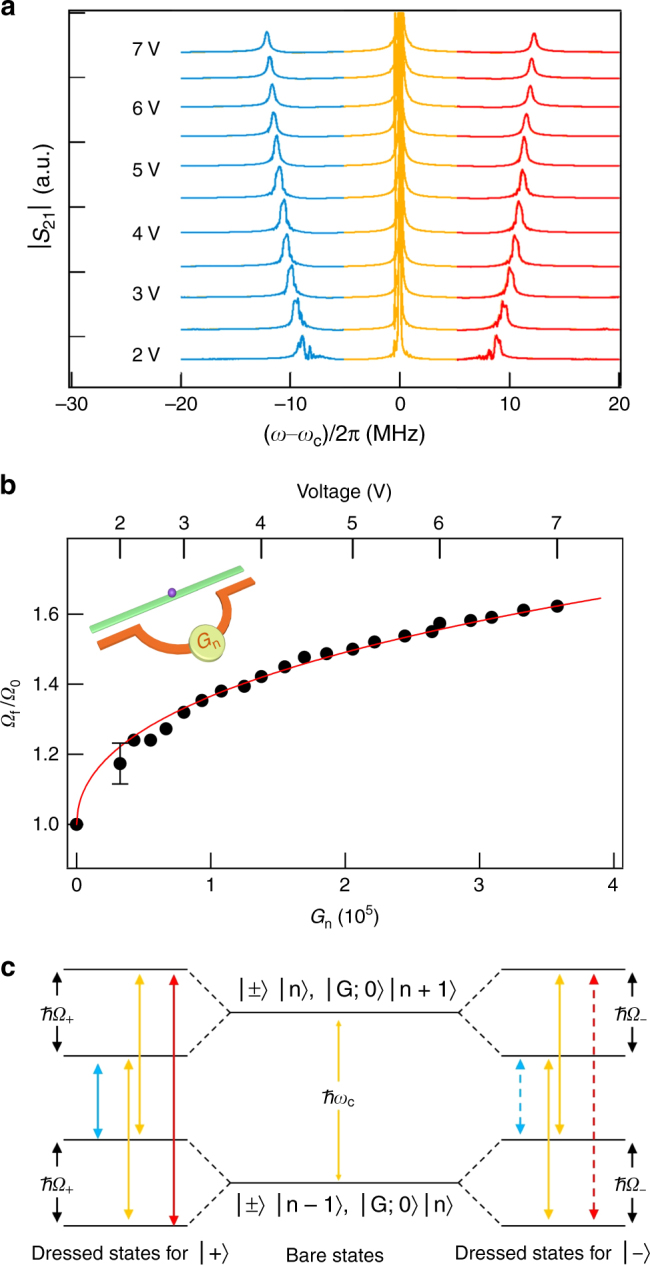
Voltage dependent Rabi frequency. **a** |*S*_21_| spectra measured at Δ = 0 and *d* = 0.58 mm (*Ω*_0_/2π = 7.5 MHz), for a series of *V*. **b** The measured (circles) and calculated (curve) ratio of Rabi frequencies *Ω*_*f*_/*Ω*_0_ plotted as a function of *V* and *G*_*n*_. The error bar is the maximum uncertainty caused by the error when fitting the Rabi frequency. **c** Schematic dressed states of both CMP modes. At Δ = 0, they produce the Mollow triplet with *Ω*_+_ = *Ω*_−_. At $\Delta \ne$0, they produce the magnon quintuplet with $\Omega _ + \ne \Omega _ -$. The detuning between the bare states is not shown for brevity

The characteristic trait of the Mollow triplet is that the Rabi splitting increases with the photon number^22, 29^. This is verified in Fig. 3a where we plot the |*S*_21_| spectra measured by changing *V*, while keeping the A-P-M device at Δ = 0 with fixed *d* = 0.58 mm (corresponding to *Ω*_0_/2*π* = 7.5 MHz). As the feedback photon number is increased by increasing *G*_*n*_, the Rabi splitting increases as shown in Fig. 3b. We find that, numerically, the measured Rabi splitting scales with *G*_*n*_ via a simple relation of $\Omega_f/ \Omega_0 = \left( {1 + \xi \sqrt {G_n} } \right)^{1/2}$ with *ξ* = 2.7 ×10^−3^. We also find that the scaling parameter *ξ* differs from device to device.

### Cooperative polariton dynamics

Now, we quantitatively explain all these results through a model which we develop to unveil the underlying physics of cooperative polariton dynamics.

Without feedback coupling, the Hamiltonian describing *N* spins collectively coupled with a cavity mode is^26^ $\hat H_0/\hbar = \omega_c\hat p^\dagger \hat p + \omega_r\hat S^z + g_0\left( {\hat S^ + \hat p + \hat p^\dagger \hat S^ - } \right)$. Here, the creation (annihilation) operators $\hat p^\dagger \left( {\hat p} \right)$ describe the P-cavity mode, $\vec S = \mathop {\sum}\nolimits_{j = 1}^N \vec S_j$ is the collective spin (magnon) variable, with each spin in YIG characterized by the operators $\hat S_j^ \pm = \hat S_j^x \pm i\hat S_j^y$ and $\hat S_j^z$.

Denoting $\left| {(S,M)} \right\rangle$ as the eigenstate of $\hat S^2$ and $\hat S^z$, and $\left| 0 \right\rangle$ as the vacuum of the radiation field, the ground state of the coupled spin-photon system^21^ is $\left| {G;0} \right\rangle = \left| {\left( {N/2, - N/2} \right);0} \right\rangle$. The excited states, denoted by $\left| {G;1} \right\rangle = \left| {\left( {N/2, - N/2} \right);1} \right\rangle$ and $\left| {E;0} \right\rangle = \left| {\left( {N/2, - N/2 + 1} \right);0} \right\rangle$, respectively, are coupled to produce two CMP states $\left| \pm \right\rangle = c_ \pm \left| {E;0} \right\rangle \pm c_ \mp \left| {G;1} \right\rangle$, where $c_ \pm = \sqrt {\left( {\Omega \pm \Delta /2} \right)/2\Omega }$ are the state amplitudes with $\Omega \equiv \sqrt {\Omega_0^2 + \left( {\Delta /2} \right)^2}$. In the CMP basis, collective excitations from $\left| {G;0} \right\rangle$ to $\left| \pm \right\rangle$ lead to the twin CMP modes appearing at $\omega _ \pm = \omega _c + \Delta /2 \pm \Omega$.

With feedback coupling, all *m* CMPs produced by magnon-photon coupling are interacting collectively with *n* feedback photons. We find that such a cooperative polariton dynamics is described by the Hamiltonian (see Supplementary Notes 4–6 for details)

2 $$\hat H/\hbar = \omega _c\hat a^\dagger \hat a + \mathop {\sum}\limits_{k = \pm } \omega _k\hat m_k^z + \mathop {\sum}\limits_{k = \pm } \frac{{c_k\Omega _0}}{{\sqrt m }}\left( {\hat m_k^ + \hat a + \hat a^\dagger \hat m_k^ - } \right),$$

where $\hat a^\dagger \left( {\hat a} \right)$ describe the feedback photons, $\hat m_ \pm ^z = \left| \pm \right\rangle \left\langle \pm \right|$, $\hat m_ \pm ^ + = \left| \pm \right\rangle \left\langle {G;0} \right|$, and $\hat m_ \pm ^ - = \left| {G;0} \right\rangle \left\langle \pm \right|$ are two-level operators written in the CMP basis.

Eq. (2) links two coherent cooperative dynamics: the collective excitation of the *N* spin ensemble by magnon-photon coupling, and the collective de-excitation of the *m* CMP ensemble due to the coupling of polaritons with the coherently fedback photons. It leads to dressed states^2^, as shown in Fig. 3c. The feedback enhanced Rabi frequency is calculated (see Supplementary Note 6) for each CMP mode by considering the coupling between the states $\left| \pm \right\rangle \left| n \right\rangle$ and $\left| {G;0} \right\rangle \left| {n + 1} \right\rangle$, and we find

3 $$\Omega _ \pm = \sqrt {\left( {\Omega \pm \Delta /2} \right)^2 + 2\left( {f\Omega _0} \right)^2\left( {\Omega \pm \Delta /2} \right)/\Omega } ,$$

where $f \equiv \sqrt {n/m}$ is the feedback factor that can be tuned by changing *G*_*n*_.

At Δ = 0, we obtain from Eq. (3) $\Omega _ \pm = \Omega _f = \Omega _0\sqrt {1 + 2f^2}$, which means that the two sets of dressed states shown in Fig. 3c have the same Rabi splitting. Therefore, they produce the same triplet at *ω*_*c*_ and *ω*_*c*_±*Ω*_*f*_, as observed in Figs. 2c and 3a. Setting *f* = 0.78 for *V* = 7 V, we obtain *Ω*_*f*_ = 1.5*Ω*_0_, which produces the dashed line in Fig. 2b.

When the gain is tuned by changing V, we have $n \propto G_n$. In the linear regime, by assuming that the number of CMP is proportional to the non-equilibrium magnetization which depends linearly on the rf field *h* ^27^, we find $m \propto \sqrt {G_n}$. Hence, Eq. (3) leads to the scaling of $\Omega _f / \Omega _0 = \left( {1 + \xi \sqrt {G_n} } \right)^{1/2}$ at Δ = 0, where ξ is a device-specific parameter. This gives the theoretical curve calculated in Fig. 3b.

Furthermore, Eq. (3) predicts that at $\Delta \ne$0, a cavity magnon triplet will split because $\Omega _ + \ne \Omega _ -$. Five modes are expected to appear at *ω*_*c*_, *ω*_*c*_±*Ω*_+_, and *ω*_*c*_±*Ω*_−_. Indeed, such an intriguing cavity magnon quintuplet, produced by the cooperative dynamics of CMP, occurs when we change the *H* field.

### Cavity magnon quintuplet

**Fig. 4:**
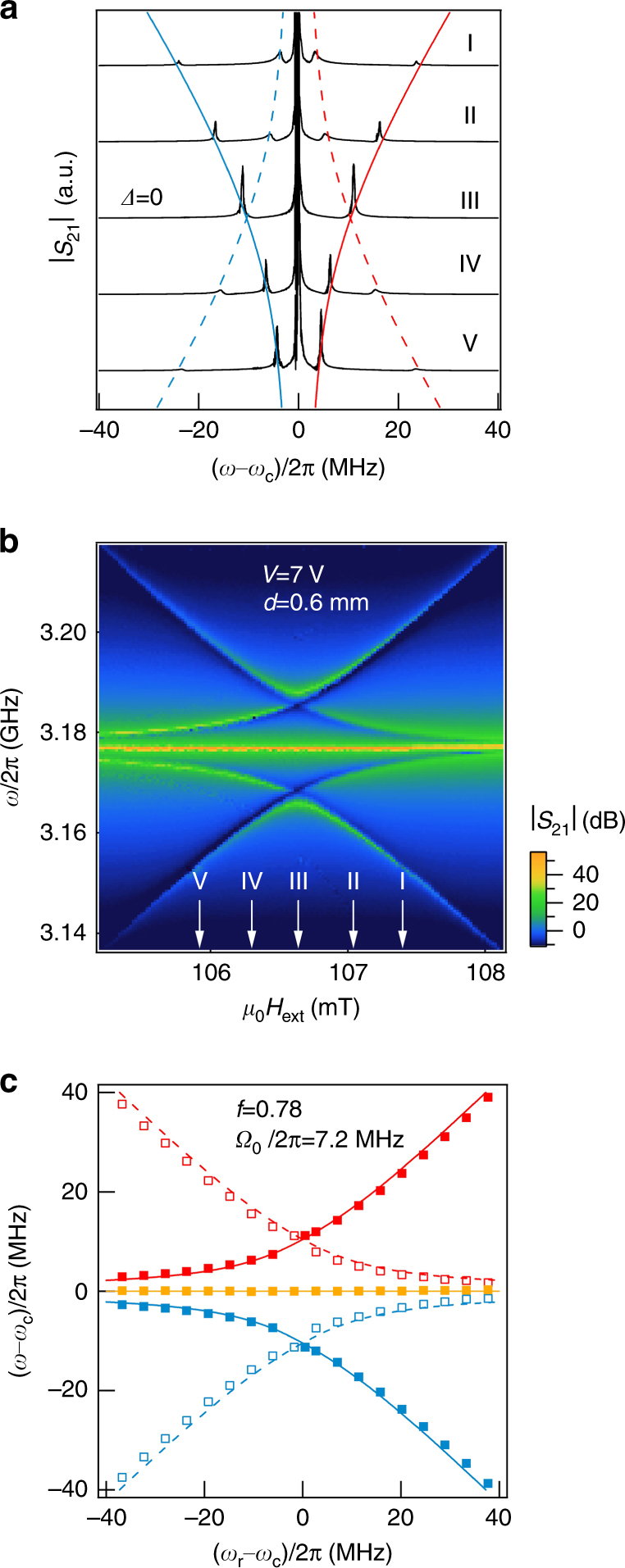
Magnetic field dependent measurement. **a** |*S*_21_| spectra measured at *d* = 0.6 mm (*Ω*_0_/2*π* = 7.2 MHz) and *V* = 7 V (*f* = 0.78), for five different *H* fields as marked in **b**. The dispersion of the measured magnon quintuplet is plotted as a function of **b**, the magnetic field *H*, and **c**, the detuning Δ/2*π*. The curves in **c** are calculated by using eq. (3)

Figure 4a shows the |*S*_21_| spectra measured at different *H*, with fixed *V* = 7 V and *d* = 0.6 mm. Clearly, when Δ is tuned away from zero, each of the modes at $\omega _ \pm / 2\pi = 3.177 \pm \Omega _f / 2\pi$ split into a doublet, and the splitting increases with |Δ|. The quintuplet dispersions are plotted in Fig. 4b, 4c as a function of *H* and Δ, respectively. Using *f* = 0.78 and *Ω*_0_/2*π* = 7.2 MHz determined from Fig. 2, we calculate the dispersions from Eq. (3) without any other parameters. The result agrees very well with the measured data, as shown in Fig. 4c.

Thus, the A-P-M devices coherently utilize two correlated aspects of cooperative dynamics in the feedback-coupled cavity systems. The first aspect, known for more than six decades as cooperative spin dynamics, is rooted in Dicke’s seminal work^23^. Signified by the Dicke factor $\sqrt N$, it describes the collective dynamics of the *N* spin system coupled with the vacuum of the radiation field. The second aspect, revealed in this work as cooperative polariton dynamics, shows the collective dynamics of *m* polaritons coupled with the higher Fock states of *n* feedback photons, which manifests itself by the feedback factor of $\sqrt {n/m}$. Using this effect, we demonstrate the breaking of harmonic protection for magnon-photon cooperativity in the linear dynamic regime ($m \ll N$), and discover the evolution of CMP to a cavity magnon triplet and a quintuplet. We note that although our experiment is based on a ferromagnetic YIG sample, the physics of cooperative polariton dynamics revealed in our study is general. With the feedback cavity technique, new avenues are in sight for harnessing the light–matter interactions at room temperature in the development of cavity spintronics and quantum technologies.

## Methods

### Experimental details

The planar microwave cavities are fabricated from Rogers RT/duroid 5880 High Frequency Laminates. The relative permittivity of the substrate is 2.2, with a loss tangent of 0.0009. The substrate is 1.57 mm thick and covered by 17.5 μm thick copper layers on both sides. An YIG sphere (M) with a diameter of 1 mm is attached to a Polytetrafluoroethylene rod which is transparent to microwaves. The rod is further connected to a stepping motor, so that the position of YIG sphere can be precisely controlled to tune the coupling strength between the YIG sphere and the cavity. All the measurements are performed at room-temperature. The cavity along with the YIG sphere as a whole hybrid system is immersed in a static external magnetic field. The *S*_21_ spectra of the hybrid system is obtained using a Vector Network Analyzer (Agilent PNA N5230C) to measure the transmission signal of the cavity from port 1 to 2 with an input microwave power of −50 dBm.

### Theoretical description

The measured *S*_21_ spectra of the A-P and P-M devices are analyzed by using input-output theory to determine the quality factor, gain, and coupling strength. The measured dispersions of the A-P-M hybrid system are quantitatively and consistently explained by developing a model based on quantum theory. Without feedback photons, *N* spins are collectively driven by the radiation field of the P-cavity mode. When the gain of the A-cavity is switched on, in addition to the magnon-photon coupling that produces *m* CMPs, the ensemble of CMP also collectively couples with the *n* coherently fedback photons. These two coherently linked cooperative dynamics are described by Eq. (2) in the main text, as well as by Supplementary Eq. 10. Based on this Hamiltonian, the quintuplet dispersions and the feedback photon enhanced Rabi frequency are calculated. In the Supplementary Notes 4–6, the theoretical derivation and the quantum picture of both magnon–photon coupling and polariton-photon coupling are explained in detail.

### Data availability

All relevant data are available from the authors.

## Electronic supplementary material

Supplementary Information

## References

[1] Haroche S (2013). Controlling photons in a box and exploring the quantum to classical boundary. Rev. Mod. Phys..

[2] Cohen-Tannoudji, C., Dupont-Roc, J. & Grynberg, G. *Atom-Photon Interactions* (Wiley-VCH, 2004).

[3] Huebl H (2013). High cooperativity in coupled microwave resonator ferrimagnetic insulator hybrids. Phys. Rev. Lett..

[4] Tabuchi Y (2014). Hybridizing ferromagnetic magnons and microwave photons in the quantum limit. Phys. Rev. Lett..

[5] Zhang X, Zou CL, Jiang L, Tang HX (2014). Strongly coupled magnons and cavity microwave photons. Phys. Rev. Lett..

[6] Goryachev M (2014). High-cooperativity cavity QED with magnons at microwave frequencies. Phys. Rev. Appl..

[7] Bai L (2015). Spin pumping in electrodynamically coupled magnon-photon systems. Phys. Rev. Lett..

[8] Haigh JA, Lambert NJ, Doherty AC, Ferguson AJ (2015). Dispersive readout of ferromagnetic resonance for strongly coupled magnons and microwave photons. Phys. Rev. B.

[9] Tabuchi Y (2015). Coherent coupling between a ferromagnetic magnon and a superconducting qubit. Science.

[10] Wang YP (2016). Magnon Kerr effect in a strongly coupled cavity-magnon system. Phys. Rev. B.

[11] Yao BM (2015). Theory and experiment on cavity magnon-polariton in the one-dimensional configuration. Phys. Rev. B.

[12] Harder M, Bai L, Match C, Sirker J, Hu CM (2016). Study of the cavity-magnon-polariton transmission line shape. Sci. China Phys. Mech. Astronomy.

[13] Cao Y, Yan P, Huebl H, Goennenwein ST, Bauer GE (2015). Exchange magnon-polaritons in microwave cavities. Phys. Rev. B.

[14] Chiorescu I, Groll N, Bertaina S, Mori T, Miyashita S (2010). Magnetic strong coupling in a spin-photon system and transition to classical regime. Phys. Rev. B.

[15] Kohn W (1961). Cyclotron resonance and de Haas-van Alphen oscillations of an interacting electron gas. Phys. Rev..

[16] Hu CM, Batke E, Köhler K, Ganser P (1995). Interaction coupled cyclotron transitions of two-dimensional electron systems in GaAs at high temperatures. Phys. Rev. Lett..

[17] Maag T (2016). Coherent cyclotron motion beyond Kohn’s theorem. Nat. Phys..

[18] Hu CM (2016). Dawn of cavity spintronics. Phys. Canada.

[19] Tabuchi Y (2016). Quantum magnonics: the magnon meets the superconducting qubit. Comp. R. Phys..

[20] Zhang X, Zou CL, Jiang L, Tang HX (2016). Cavity magnomechanics. Sci. Adv..

[21] Soykal OumlO, Flatté ME (2010). Strong field interactions between a nanomagnet and a photonic cavity. Phys. Rev. Lett..

[22] Xu X (2007). Coherent optical spectroscopy of a strongly driven quantum dot. Science.

[23] Dicke RH (1954). Coherence in spontaneous radiation processes. Phys. Rev..

[24] Scheibner M, Schmidt T, Worschech L, Forchel A, Bacher G, Passow T, Hommel D (2007). Superradiance of quantum dots. Nat. Phys..

[25] Noe GT (2012). Giant superfluorescent bursts from a semiconductor magneto-plasma. Nat. Phys..

[26] Agarwal GS (1984). Vacuum-field Rabi splittings in microwave absorption by Rydberg atoms in a cavity. Phys. Rev. Lett..

[27] Gui YS, Wirthmann A, Mecking N, Hu CM (2009). Direct measurement of nonlinear ferromagnetic damping via the intrinsic foldover effect. Phys. Rev. B.

[28] Wang, Y.-P., Zhang, D., Li, T.-F., Hu, C.-M., & You, J. Q. Bistability of Cavity Magnon-Polaritons. Preprint at http://arxiv.org/abs/1707.06509v1 (2017). 10.1103/PhysRevLett.120.057202 29481165

[29] Mollow BR (1969). Power spectrum of light scattered by two-level systems. Phys. Rev.

