# Supplementary material for: Cooperative polariton dynamics in feedback-coupled cavities
Source: Nat Commun. 2017 Nov 10;8:1437. doi: 10.1038/s41467-017-01796-7 (PMC5681655; doi:10.1038/s41467-017-01796-7)
Supplement: Supplementary file 1 — Supplementary Information [file 41467_2017_1796_MOESM1_ESM.pdf]

Supplementary Note 1: Design of the resonators

Using the feedback-coupled resonators to study magnon-photon coupling is the key technical innovation of this work. The resonators are designed by using the CST simulation and fabricated from RT/duroid 5880 High Frequency Laminates. The relative permittivity of the substrate is 2.2, with a loss tangent of 0.0009. The substrate is 1.57 mm thick and covered by a 17.5 μm copper layers on both sides. The passive resonator (P) as well as the active resonator (A) are designed with their width set at 1.5 mm. A voltage controlled microwave amplifier is inserted into the planar resonant A to realize the gain. Both the resonators A and P are designed to operate at the same frequency at the half-wavelength mode with a phase shift π, which makes a total phase shift of 2π in the loop. This ensures that with the gain, a constructive feedback is created in the cavity, which compensates the power loss and hence the high-quality factor cavity is obtained. A detailed microwave circuit layout of the device is shown in Supplementary Fig. 1.

In this figure, the P cavity, the A cavity, the Magnons and the Amplifier are shown by their LCR (inductor-capacitor-resistor) circuit layouts. Two AC blocks (RF chokes) were added to decouple the microwave circuit from the DC bias circuit. In the Supplementary

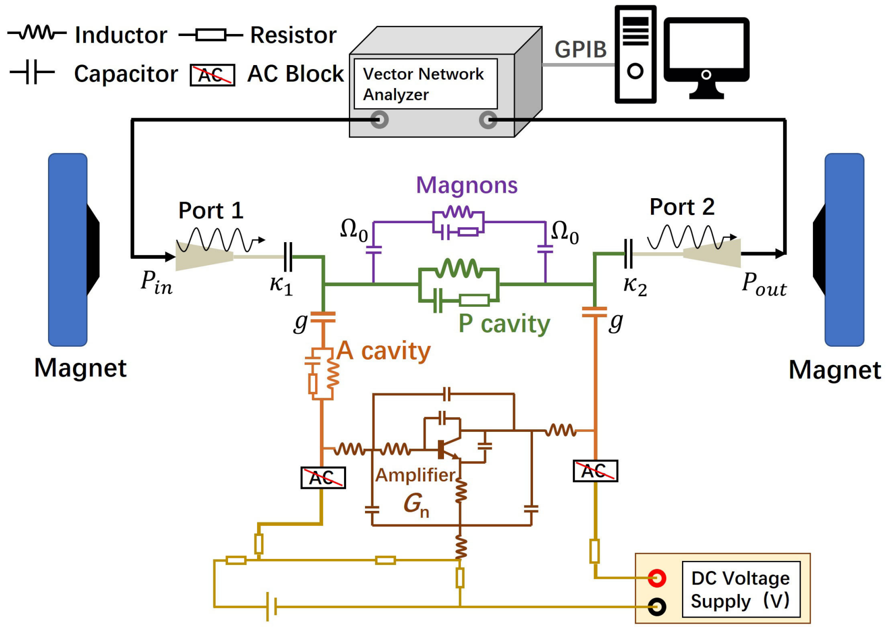

Supplementary Figure 1 **Layout of the device structure and experimental setup** The P cavity, the A cavity, the Amplifier and Magnons are shown by their LCR circuits.

Notes 2 and 3, measurement results for the A cavity and the A-P coupled cavities are discussed in detail, respectively.

Supplementary Note 2: Characterization of the A resonator

The resonant nature of the feedback resonator A is verified by direct measurement of its resonance frequency and quality factors. For the purpose of such a characterization, we have fabricated a stand-alone resonator that is not coupled to the resonator P, which has an identical geometrical design of the one that is coupled to the P resonator.

Supplementary Fig. 2 shows the measured $|S_{21}|$ spectra of resonator A at different bias voltages. The spectra are analysed by using the input-output theory, with

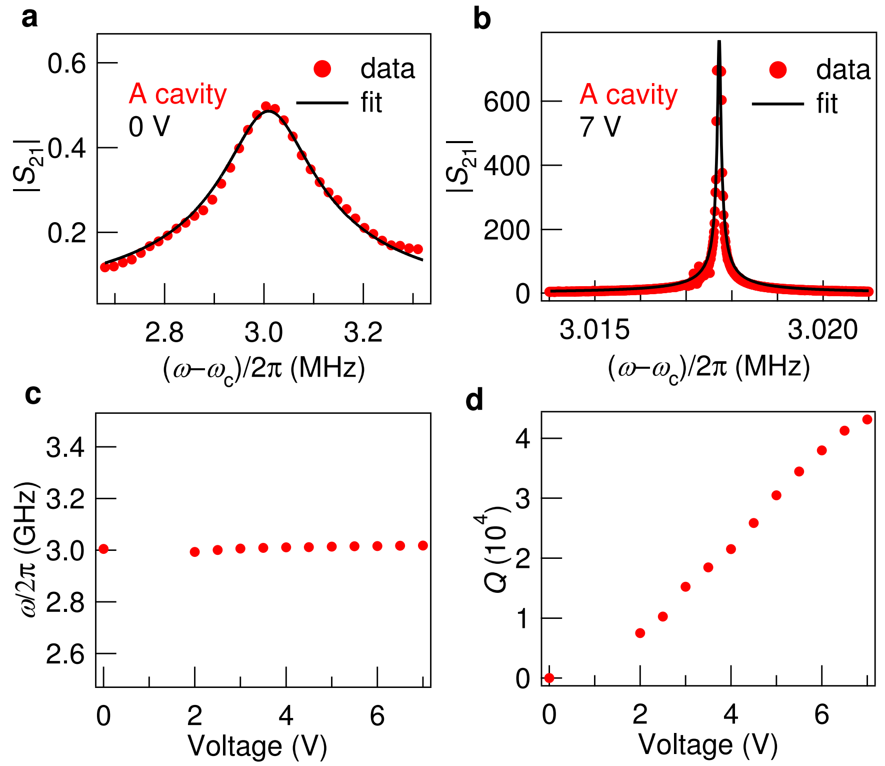

Supplementary Figure 2 **Characterization of the A resonator**

a, Measured $|S_{21}|$ of the A cavity with the Supplementary Equation (1) fit at V=0 V.

b, Measured $|S_{21}|$ of the A cavity with the Supplementary Equation (1) fit at V=7 V.

c, Measured A cavity resonance frequency as a function of voltage.

d, Measured Q factor of A-cavity as a function of voltage.

$$|S_{21}| = \left| \frac{\sqrt{\kappa_1 \kappa_2}}{(\omega - \omega_c) + \frac{i(\kappa_A + g_A)}{2}} \right|, \quad (1)$$

where $\kappa_A > 0$ is the intrinsic damping parameter of the resonator A at zero voltage, $g_A < 0$ is the voltage dependent gain parameter of the resonator A, and $\kappa_1 = \kappa_2$ is the coupling rate at port 1 and 2, respectively. From the fit, we determine the resonance frequency $\omega_c/2\pi = 3$ GHz and the quality factor $Q = 17$ at $V = 0$ V, as shown in Supplementary Fig. 2a. The voltage dependences of the resonance frequency and quality factor are measured and displayed in Supplementary Figs. 2c and 2d, respectively. By the tuning of the voltage, $Q = \omega_c/(\kappa_A + g_A)$ is increased up to 4×10^4 while the resonance frequency remains stable with a deviation of less than 1%.

Supplementary Note 3: Quantifying the coupling between A and P resonators

The design requirements of resonator A and P are described in Supplementary Note 1. We set the two resonators to work at the weak coupling regime, which allows their dissipation to be coupled while operating at the nearly same frequency. The measured $|S_{21}|$ spectra of the resonators before and after coupling are shown in Supplementary Fig. 3.

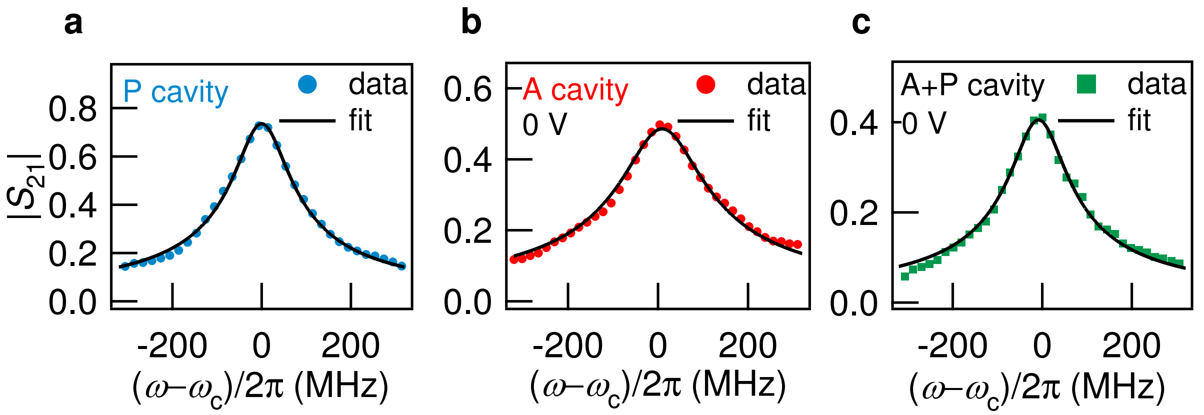

Supplementary Figure 3 **Quantifying the coupling between A and P resonators**
a, Measured $|S_{21}|$ of the P cavity with the fit by Supplementary Equation (1).
b, Measured $|S_{21}|$ of the A cavity with the fit by Supplementary Equation (1) at V=0 V.
c, Measured $|S_{21}|$ of A+P cavity with the fit by Supplementary Equation (2) at V=0 V.

The effect of the coupling can be determined by using the input-output theory for two coupled oscillators:

$$|S_{21}| = \left| \frac{\sqrt{\kappa_1 \kappa_2}}{\omega - \omega_c - \delta\omega + \frac{i\kappa_P}{2} - \frac{g^2}{\omega - \omega_c - \delta\omega + \frac{i\kappa_A}{2}}} \right|, \quad (2)$$

where κ_P and κ_A are the intrinsic damping parameters of the resonator P and A, respectively, and $\kappa_1 = \kappa_2$ is the energy loss rate at port 1 and port 2, respectively. $\delta\omega$ accounts for a small mode frequency shift due to the change of effective dielectric constant when the two cavities are brought together. By using $|S_{21}| = |\sqrt{\kappa_1\kappa_2}/(\omega - \omega_c + \frac{i\kappa_{P,A}}{2})|$ to fit the spectra measured on the two resonators separately as shown in Supplementary Figs. 3a and 3b, we determine the intrinsic damping parameters $\kappa_P = 126$ MHz and $\kappa_A = 180$ MHz. By using Supplementary Equation (2) to fit the measured spectrum of the A-P coupled cavity as shown in Supplementary Fig. 3c, we determined $\kappa_1/2\pi$=$\kappa_2/2\pi$ = 25 MHz, $\delta\omega = 160$ MHz, and the coupling parameter is estimated as $g/2\pi \ll$1 MHz. Such a weak coupling does not lift the mode degeneracy, but leads to a coupled mode with a mixed damping rate that depends on both κ_P and κ_A.

Supplementary Note 4: Theoretical description of magnon-photon coupling: general consideration

An YIG sphere is usually described as a many-body system with N spins $\vec{S}_j, (j = 1, \cdots N)$. The total spin of the sphere, $\vec{S} = \sum_{j=1}^{N} \vec{S}_j$, is the spin observable that couples to the magnetic field of a cavity, which is approximately homogeneous across a 1 mm diameter YIG sphere. Such a collective coupling of two-level systems to a cavity mode resembles Dicke's model of superradiance [1].

Agarwal [2] discovered that in the evaluation of the absorption spectra of such a coupled system in the linear dynamic regime, one only needs the eigenfunctions of a reduced space defined by the combined states $|G;0\rangle = |(\frac{N}{2}, -\frac{N}{2});0\rangle$, $|G;1\rangle = |(\frac{N}{2}, -\frac{N}{2});1\rangle$ and $|E;0\rangle = |(\frac{N}{2}, -\frac{N}{2}+1);0\rangle$, where $|(S,M)\rangle$ represents the collective spin eigenstate of $\hat{S}^2$ and $\hat{S}^z$, and $|n\rangle$ represents the photon Fock states. Using these states and by setting the energy of $|G;0\rangle$ as zero, we obtain the Hamiltonian for the A-P-M devices:

$$\hat{H}/\hbar = \omega_r \hat{m}^z + \omega_c \hat{p}^\dagger \hat{p} + \Omega_{PM}(\hat{m}^+ \hat{p} + \hat{p}^\dagger \hat{m}^-) + \omega_c \hat{a}^\dagger \hat{a} + \Omega_{APM}(\hat{m}^+ \hat{a} + \hat{a}^\dagger \hat{m}^-), \quad (3)$$

where the creation (annihilation) operators $\hat{p}^\dagger(\hat{p})$ describe the P-cavity mode, $\hat{a}^\dagger(\hat{a})$ describe the feedback photon of the A-cavity mode, $\hat{m}^z = |E;0\rangle\langle E;0|$, $\hat{m}^+ = |E;0\rangle\langle G;0|$, and $\hat{m}^- = |G;0\rangle\langle E;0|$ are two-level operators in the reduced space of Agarwal where the processes of creating and annihilating a magnon take place, $\hbar\Omega_{PM}$ and $\hbar\Omega_{APM}$ are renormalized coupling strengths for P-M and A-P-M coupling, respectively.

Supplementary Equation (3) describes the collective dynamics of a two-level system of $|G\rangle$ and $|E\rangle$, which are coupled to two radiation fields. We calculate $\hbar\Omega_{PM}$ and $\hbar\Omega_{APM}$ by using the collective spin state $|(\frac{N}{2}, -\frac{N}{2}+m)\rangle$ that involves m spin excitations, and average the obtained coupling energy over the m collective spin excitations by using the mean-field approach

$$\langle\Omega_m\rangle = \frac{1}{m}[\langle(\frac{N}{2}, -\frac{N}{2}+m);0|g_0(\hat{S}^+\hat{a}+\hat{a}^\dagger\hat{S}^-)|(\frac{N}{2}, -\frac{N}{2}+m-1);1\rangle] = \sqrt{\frac{N-m+1}{m}}g_0. \quad (4)$$

Here, g_0 is the vacuum Rabi frequency of the single spin as defined in the main text. Note that in Supplementary Equation (4), we are consistently treating the N spins and m spin excitations as a quantum-mechanical system. In the limit of linear spin dynamic regime with $m \ll N$, the Dicke factor $\sqrt{N}$ naturally appears in Supplementary Equation (4) as the matrix element of the raising operator $\hat{S}^+$ for all N spins. It describes the celebrated physics of N spins collectively coupled with the cavity photons [1, 2]. In addition, another factor $\sqrt{m}$ appears due to the m collective spin excitations. As we explain in the following sections, it reveals the hidden physics of m CMPs collectively coupled with the feedback cavity photons. In our A-P-M devices, these two collective dynamic effects are coherently linked to enable the breaking of harmonic protection of magnon Rabi frequency.

Supplementary Note 5: Theoretical description of CMP: Magnon-photon coupling in the P-M device

Without the A-cavity, Supplementary Equation (3) reduces to

$$\hat{H}_0/\hbar = \omega_r\hat{m}^z + \omega_c\hat{p}^\dagger\hat{p} + \Omega_{PM}(\hat{m}^+\hat{p} + \hat{p}^\dagger\hat{m}^-), \quad (5)$$

which describes the magnon-photon coupling in the P-M device. It creates the cavity magnon polariton (CMP). Note that in the linear dynamic regime where the number of CMP $m \ll N$, the many-spin system is far from being saturated by the photon excitation, so that adding

photons may increase m but does not enhance the coupling strength Ω_{PM}. Therefore, the P-M coupling in the reduced space of Agarwal can be simply described by the elementary process of creating one CMP (m =1) from the ground state $|G;0\rangle$, and we get from Supplementary Equation (4)

$$\Omega_{PM} = \langle \Omega_1 \rangle = \sqrt{N} g_0 = \Omega_0. \tag{6}$$

From Supplementary Equation (5) at the detuning $\Delta = \omega_r - \omega_c$, we get the CMP eigenstates

$$|\pm\rangle = c_{\pm}|E;0\rangle \pm c_{\mp}|G;1\rangle, \tag{7}$$

with the eigenfrequencies

$$\omega_{\pm} = \omega_c + \frac{\Delta}{2} \pm \Omega, \tag{8}$$

where $c_{\pm} = \sqrt{(\Omega \pm \Delta/2)/2\Omega}$ are the state amplitudes and $\Omega \equiv \sqrt{\Omega_0^2 + (\Delta/2)^2}$. Our result is consistent with Agarwal's theory for N atoms [2], and it is also in agreement with the CMP theory set on the footing of coupled harmonic oscillators [3].

Supplementary Note 6: Theoretical description of cavity magnon quintuplet: polariton-photon coupling in the A-P-M device

With the A-cavity, in addition to the collective excitation of CMP due to magnon-photon coupling, we must also consider the collective effect of m CMPs coupled with the coherently feedback radiation field. Taking into account such a cooperative effect by using Supplementary Equation (4) at the linear dynamic regime ($1 \ll m \ll N$), we find the renormalized A-P-M coupling strength in the reduced space

$$\Omega_{APM} = \langle \Omega_m \rangle \simeq \sqrt{\frac{N}{m}} g_0 = \frac{\Omega_0}{\sqrt{m}}. \tag{9}$$

Since apparently $\Omega_{APM} \ll \Omega_{PM}$, we diagonalize H_0 of Supplementary Equation (5) by using the CMP wave function $|\pm\rangle$ of Supplementary Equation (7), and we obtain $H/\hbar = \omega_+ \hat{m}_+^z + \omega_- \hat{m}_-^z + \omega_c \hat{a}^\dagger \hat{a} + \Omega_{APM}(\hat{m}^+ \hat{a} + \hat{a}^\dagger \hat{m}^-)$ from Supplementary Equation (3), where $\hat{m}_\pm^z = |\pm\rangle\langle\pm|$ are the projection operators for the CMP states. Furthermore, by using the reverse transformation of Supplementary Equation (7) to replace $|E;0\rangle$ with $|\pm\rangle$ in the operators $\hat{m}^\pm$, we transform the reduced space of Agarwal to the new reduced space of CMP

composed of the states $|G;0\rangle$ and $|\pm\rangle$, in which we find the Hamiltonian for the A-P-M device

$$\hat{H}/\hbar = \omega_+ \hat{m}_+^z + \omega_- \hat{m}_-^z + \omega_c \hat{a}^\dagger \hat{a} + \frac{c_+ \Omega_0}{\sqrt{m}}(\hat{m}_+^+ \hat{a} + \hat{a}^\dagger \hat{m}_+^-) + \frac{c_- \Omega_0}{\sqrt{m}}(\hat{m}_-^+ \hat{a} + \hat{a}^\dagger \hat{m}_-^-), \tag{10}$$

where $\hat{m}_\pm^+ = |\pm\rangle\langle G;0|$ and $\hat{m}_\pm^- = |G;0\rangle\langle\pm|$ are two-level operators for the elementary process of creating and annihilating a CMP.

Supplementary Equation (10) consistently treats the P-M and A-P-M coupling in the reduced space of CMP, which describes two coherently linked cooperative dynamics: the collective excitation of N spins via the magnon-photon coupling, and the collective de-excitation of m CMPs via the polariton-feedback-photon coupling. It leads to the dressed states of each CMP mode, whose Rabi frequency $\Omega_\pm$ can be calculated by considering the coupling between the combined CMP-photon states $|\pm\rangle|n\rangle$ and $|G;0\rangle|n+1\rangle$. We find

$$\Omega_\pm = \sqrt{(\Omega \pm \Delta/2)^2 + 2(f\Omega_0)^2(\Omega \pm \Delta/2)/\Omega}, \tag{11}$$

where in addition to the Dicke factor of $\sqrt{N}$ that appears in Ω_0, a new feedback factor emerges with

$$f = \sqrt{\frac{n}{m}}. \tag{12}$$

Physically, f denotes the de-excitation ratio of the CMPs, and it is the signature of cooperative polariton dynamics involving collective coupling of m CMPs with n feedback photons. Experimentally by using the A-P-M device, f is determined by the gain of the device as explained in the main text, which can be tuned by changing the voltage.

At $\Delta = 0$, we get from Supplementary Equation (11) that $\Omega_\pm = \Omega_f = \Omega_0\sqrt{1+2f^2}$, which produces cavity magnon triplet at ω_c and $\omega_c \pm \Omega_f$. At $\Delta \neq 0$, the triplet splits because $\Omega_+ \neq \Omega_-$, which leads to the cavity magnon quintuplet appearing at ω_c, $\omega_c \pm \Omega_+$, and $\omega_c \pm \Omega_-$.

Supplementary references

[1] Dicke, R. H. Coherence in spontaneous radiation processes. *Phys. Rev.* **93**, 99 (1954).

[2] Agarwal, G. S. Vacuum-field Rabi splittings in microwave absorption by Rydberg atoms in a cavity. *Phys. Rev. Lett.* **53**, 1732 (1984).

[3] Harder, M., Bai, L., Match, C., Sirker, J. & Hu, C.-M. Study of the cavity-magnon-polariton transmission line shape. *Science China Physics, Mechanics & Astronomy* **59**, 117511 (2016).
